# Cost-effectiveness of general practitioner- versus surgeon-led colon cancer survivorship care: an economic evaluation alongside a randomised controlled trial

**DOI:** 10.1007/s11764-023-01383-4

**Published:** 2023-04-25

**Authors:** Julien A. M. Vos, Mohamed El Alili, Laura A. M. Duineveld, Thijs Wieldraaijer, Jan Wind, Edanur Sert, Sandra C. Donkervoort, Marc J. P. M. Govaert, Nanette A. W. van Geloven, Anthony W. H. van de Ven, Gijsbert Heuff, Henk C. P. M. van Weert, Judith E. Bosmans, Kristel M. van Asselt, on behalf of the ICARE study group

**Affiliations:** 1 Department of General Practice, Amsterdam UMC, University of Amsterdam, Meibergdreef 9, 1105 AZ Amsterdam, the Netherlands; 2 Amsterdam Public Health Research Institute, Amsterdam, the Netherlands; 3 Department of Health Sciences, Faculty of Science, Vrije Universiteit Amsterdam, Van Der Boechorstraat 7, 1081 BT Amsterdam, the Netherlands; 4 Department of Surgery, OLVG Hospital, Oosterpark 9, 1091 AC Amsterdam, the Netherlands; 5 Department of Surgery, Dijklander Hospital, Maelsonstraat 3, 1624 NP Hoorn, the Netherlands; 6 Department of Surgery, Tergooi Hospital, Van Riebeeckweg 212, 1213 XZ Hilversum, the Netherlands; 7 Department of Surgery, Flevoziekenhuis, Hospitaalweg 1, 1315 RA Almere, the Netherlands; 8 Department of Surgery, Spaarne Gasthuis, Spaarnepoort 1, 2134 TM Hoofddorp, the Netherlands

**Keywords:** Colon cancer, Primary health care, Quality of healthcare, Cancer survivors, Cost–benefit analysis

## Abstract

**Purpose:**

The aim of this study is to assess cost-effectiveness of general practitioner (GP) versus surgeon-led colon cancer survivorship care from a societal perspective.

**Methods:**

We performed an economic evaluation alongside the I CARE study, which included 303 cancer patients (stages I–III) who were randomised to survivorship care by a GP or surgeon. Questionnaires were administered at baseline, 3-, 6-, 12-, 24- and 36-months. Costs included healthcare costs (measured by iMTA MCQ) and lost productivity costs (SF-HLQ). Disease-specific quality of life (QoL) was measured using EORTC QLQ-C30 summary score and general QoL using EQ-5D-3L quality-adjusted life years (QALYs). Missing data were imputed. Incremental cost-effectiveness ratios (ICERs) were calculated to relate costs to effects on QoL. Statistical uncertainty was estimated using bootstrapping.

**Results:**

Total societal costs of GP-led care were significantly lower compared to surgeon-led care (mean difference of − €3895; 95% CI − €6113; − €1712). Lost productivity was the main contributor to the difference in societal costs (− €3305; 95% CI − €5028; − €1739). The difference in QLQ-C30 summary score over time between groups was 1.33 (95% CI − 0.049; 3.15). The ICER for QLQ-C30 was − 2073, indicating that GP-led care is dominant over surgeon-led care. The difference in QALYs was − 0.021 (95% CI − 0.083; 0.040) resulting in an ICER of 129,164.

**Conclusions:**

GP-led care is likely to be cost-effective for disease-specific QoL, but not for general QoL.

**Implications for cancer survivors:**

With a growing number of cancer survivors, GP-led survivorship care could help to alleviate some of the burden on more expensive secondary healthcare services.

## Introduction

In the coming decades, the incidence of colon cancer is expected to rise globally [1]. Patients who have been curatively treated for colon cancer enter a survivorship care program. Survivorship care involves surveillance of possible recurrences and also attention to medical and psychosocial late effects of cancer and its treatment [2]. In most countries, survivorship care is provided by a specialist at the outpatient clinic. The growing number of patients in need of survivorship care is putting additional demands on current, hospital-based healthcare services [1, 3]. In 2019, the Dutch national healthcare costs related to colon cancer were estimated at 558 million euros, which included both treatment and survivorship care costs [4]. This accounted for approximately 9% of all healthcare costs relating to cancer (6.5 billion). Since hospital-based care is costly [5], it is relevant to investigate other and potentially more cost-effective strategies, such as care by a general practitioner (GP).

Cancer patients frequently contact their GP for problems relating to cancer and its treatment. In the first years after a colon cancer diagnosis, primary healthcare use is markedly increased compared to the years before the cancer diagnosis [6, 7]. GPs are trained to provide patient-centered, rather than disease-focused care, and provide care in the context of other physical, emotional and social needs. The patient-centered approach of the GP could increase the quality of survivorship care [8]. It has therefore been suggested that survivorship care could be provided by a GP instead of a specialist [9]. Active involvement of the GP is also asked for by patients [10]. Clinical and patient-reported outcomes of survivorship care by the GP are similar to those by a specialist, while it can reduce healthcare costs [11, 12]. However, the level of evidence regarding this topic is generally low due to the limited number and quality of studies.

The I CARE study (Improving Care After colon canceR treatment in the Netherlands, personalised care to Enhance quality of life) is a randomised controlled trial comparing colon cancer survivorship care by a GP to care by a surgeon (usual care) [13]. Within the first year after surgery, there were no clinically meaningful differences in quality of life (QoL) between the two groups [14]. Other outcomes, including costs, are important to take into consideration [15]. Therefore, we performed an economic evaluation alongside the I CARE study comparing GP-led to surgeon-led survivorship care from a societal perspective. We hypothesized that survivorship care by a GP is cost-effective in comparison to surgeon-led survivorship care.

## Methods

### Trial

#### Trial design and setting

The I CARE study is an ongoing 2 × 2 factorial randomised-controlled trial, comparing colon cancer survivorship care by a GP to care by a surgeon, with or without access to Oncokompas, a supporting eHealth application [13]. By the end of 2023, all patients will have finished their 5-year follow-up period [16]. The I CARE study is conducted in eight hospitals in the Netherlands. Patients who have been curatively treated for stage I–III colon cancer were considered eligible for the study. The guideline for colorectal cancer was summarised into a survivorship care plan and provided to the participating GPs [17]. The recommended follow-up schedule was identical in both trial arms. Quality of life (QoL) was the primary outcome. Cost-effectiveness of GP-led care was a secondary research objective.

#### Trial procedures

Multiple questionnaires were sent out; at baseline (shortly after randomisation), at 3, 6, 12, 24, 36, 48 and 60 months after surgery. Castor EDC was used to enter baseline and questionnaire data [18]. Depending on the tumour stage, patients go for routine check-ups every 3- to 6-months within the first 3 years after surgery, and once a year thereafter [17]. For the economic evaluation, the protocol pre-specified a time horizon up to 5 years after surgery [13]. However, most recurrences (> 90%) are detected within the first 3 years after surgery [19] and the frequency of check-up decreases thereafter [17]. Because most differences are expected within this time period, we restricted the analyses to 3 years of follow-up.

### Outcome measures

#### Effect outcomes

For the economic evaluation, two main effect outcomes were used. Disease-specific QoL was measured using the EORTC QLQ-C30 summary score for QoL [20] and general QoL using the three-level version of the EuroQol instrument (EQ-5D-3L) [21]. The QLQ-C30 summary score has a range from 0 to 100 with higher scores indicating better quality of life [20, 22]. Overall quality of life was measured using the EQ-5D-3L [21]. The Dutch EQ-5D-3L tariff was used to convert EQ-5D-3L health states to utility scores [23]. QALYs were then calculated by multiplying the utility score of a specific health state with the time spent in that health state. Effect outcomes after the first year were discounted using a discount rate of 1.5% [5].

#### Cost outcomes

Costs were measured from a societal perspective, meaning that both healthcare costs and lost productivity costs were taken into account. The iMTA Medical Consumption Questionnaire (iMCQ) was used to assess healthcare utilisation [24]. Healthcare utilisation was valued using standard prices from the Dutch costing guideline [5]. Healthcare utilisation included different types of visits to healthcare providers (primary and secondary care, and emergency visits), day and home care, admissions, and medication. Medication was classified into categories based on the mechanisms of action. For each category, an average price was calculated using pricing data from the Dutch National Health Care Institute [25].

Productivity losses were measured using the Short-Form Health and Labour Questionnaire (SF-HLQ) [26]. Absenteeism costs from paid work were calculated using the friction cost approach (FCA). The FCA assumes that sick employees get replaced after a certain point in time (the friction period) and that, consequently, there is no productivity loss anymore. We used a friction period of 102 days. Absenteeism costs from unpaid work were calculated using a shadow price for providing informal care. Presenteeism, which is defined as a reduced efficiency at work due to health-related problems, was calculated by multiplying (1-efficiency score) with the number of hours that the patient was suffering from health-related problems. Gender-specific estimates of the mean wages of the Dutch population were used to calculate the lost productivity costs related to paid work[5]. Costs after the first year were discounted using a discount rate of 4% [5].

### Statistical analyses

#### Missing data

The cost-effectiveness analyses were conducted according to the intention-to-treat principle. Missing data were imputed using multiple imputation with chained equations (MICE) [27]. Cost and effect data were assumed to be missing at random, which means that missing observations are explained by observed variables [28]. The imputation model included outcome variables and predictor variables that differed at baseline, were related to missing data or were associated with the outcome (see Table 2 for variables included in imputation model). To account for the skewed distribution of cost data, predictive mean matching was used in MICE [29]. The number of imputed datasets was increased until the loss of efficiency was less than 5%, resulting in 5 imputed datasets [29]. Each of the imputed datasets was analysed separately as described below. Results from the multiple datasets were pooled using Rubin’s rules [30].

#### Cost-effectiveness and cost-utility analyses

Ordinary least squares regression was used to estimate incremental costs and effects between the treatment groups. However, for the QLQ-C30 summary score, a mixed model was used. Hence, adjustment for the longitudinal nature of the data took place. This was done by specifying a two-level structure where repeated patients’ observations (i.e. patients’ QLQ-C30 summary scores at different time points) were nested within patients. This was implemented by allowing the intercepts to vary between clusters (i.e. random intercepts model) [31, 32] which allowed for estimation of an overall effect over time [33]. In addition, the overall effect over time was adjusted for baseline QLQ-C30 scores by omitting the baseline QLQ-C30 summary score from the mixed model [34]. QALYs were adjusted for baseline utilities [35]. Costs were adjusted for utilities and QLQ-C30 summary score at baseline. Incremental cost-effectiveness ratios (ICERs) were calculated by dividing the incremental costs by the incremental effects. Bias-corrected bootstrapping was used to estimate statistical uncertainty (5000 replications). Statistical uncertainty surrounding ICERs was illustrated by plotting the bootstrapped cost-effect pairs on a cost-effectiveness plane (CE-plane). Cost-effectiveness acceptability curves (CEACs) were also estimated, which demonstrate the probability that the intervention is cost-effective compared to usual care for a range of different ceiling ratios (i.e. the willingness-to-pay threshold for one point effect extra) [36]. CEACs were estimated using the parametric normal-based approach for incremental net-monetary benefits (INMBs) [37]. In the Netherlands, the willingness-to-pay threshold for healthcare interventions is based on disease severity [38]. For disease severities between 0.1 and 0.4, which includes colon cancer, the reference value is 20,000 € per QALY gained. For outcome measures such as the QLQ-C30 summary score, no formal willingness-to-pay threshold has been determined. Analyses were performed in StataSE 17® (StataCorp LP, CollegeStation, TX, US).

#### Sensitivity analyses

To check the robustness of the results, seven sensitivity analyses were performed. To assess impact of imputing missing data, the economic evaluation was performed on complete cases only (SA1). In countries like the UK, the healthcare perspective is used when deciding about the reimbursement of new health interventions. Therefore, the economic evaluation was also performed using a healthcare perspective, i.e. excluding lost productivity costs (SA2). To assess impact of discounting, the economic evaluation was performed without discounting future costs and QALYs (SA3). To assess impact of adjusting for covariates, unadjusted regression models were used to estimate differential costs and effects (SA4). To assess whether cost-effectiveness differed from patients who had transferred between trial arms, a per-protocol analysis was performed (SA5). Finally, because follow-up of stage I versus stage II/III colon cancer differs [17], two subgroup analyses were performed. One analysis used data from patients with stage I cancer (SA6) and a second analysis data from patients with stage II/III cancer (SA7).

## Results

The study population included 303 participants, of which 141 were randomised to the GP and 162 to the surgeon. The study population had a mean age of 68.0 years (SD 8.4), and included more males (67%) than females (Table 1). Employment did not differ between the two groups (27% in the GP-led versus 31% in the surgeon-led group). Patients in the GP-led group relatively often had stage I tumours compared to the surgeon-led group (42% versus 33%). In 22% of all cases, patients were treated with adjuvant chemotherapy. During the 3-year follow-up period, 50 patients transferred from the GP back to the surgeon. In most cases, this was due to (suspected) recurrences (*n* = 22) or patients’ preferences (*n* = 21).

**Table 1:** Baseline characteristics of the participants

	Surgeon-led care (*n* = 162)	General practitioner-led care (*n* = 141)
Sociodemographic		
Age (years, mean, SD)	68.2 (8.4)	67.9 (8.3)
Sex (male, %)	105 (65)	98 (70)
Living situation, together (%)	120 (74)	107 (76)
Educational attainment (%)		
- Primary or non	13 (8)	14 (10)
- Secondary	40 (25)	28 (20)
- Vocational education	71 (44)	75 (53)
- University	24 (15)	12 (9)
- Missing	14 (9)	12 (9)
Employed (%)	50 (31)	38 (27)
Randomised to Oncokompas (%)	83 (51)	68 (48)
Clinical and pathological		
Comorbidities (%);		
- 0 1	84 (52)	63 (45)
- ≥ 2	78 (48)	78 (55)
Cancer diagnosis via (%)		
- Population screening	78 (48)	74 (53)
- Clinical course	84 (52)	67 (48)
Tumour stage (%);		
- I	54 (33)	59 (42)
- II	54 (33)	50 (36)
- III	54 (33)	32 (23)
Stoma (%)	7 (4)	6 (4)
Chemotherapy (%)	41 (25)	27 (19)
Time between surgery and inclusion (months, median, IQR)	3.5 (1.8–6.1)	3.6 (1.8–5.9)

*SD* standard deviation, *IQR* interquartile range

### Costs

For most cost categories, costs were lower in the GP-led versus surgeon-led group (see Table 2). Day care costs and lost productivity costs in the GP-led group were significantly lower compared to the surgeon-led group. The largest difference in costs between the two groups was found for lost productivity costs (i.e. − €3305; 95% CI − €5028; − €1739), while the smallest difference was found for hospital costs (i.e. − €8; 95% CI − €866; €938). Total societal costs were €3895 lower in the GP-led group compared to the surgeon-led group, which was statistically significant (95% CI − 6113; − 1712). When adjusted for utility and QLQ-C30 summary score at baseline, total societal costs were €2759 lower in the GP-led versus surgeon-led group; this difference was also statistically significant (95% CI − €4855; − €557) (Table 3).

**Table 2:** Multiply imputed effects and costs

		Surgeon-led (*N* = 162)	General practitioner-led (*N* = 141)	
Outcomes		Mean (SE)		Mean difference (95% CI)^a^
EORTC QLQ-C30 summary score	T0 (baseline) T1 (3 months) T2 (6 months) T3 (12 months) T4 (24 months) T5 (36 months)	86.15 (0.87) 88.92 (0.98) 88.90 (0.91) 90.32 (0.89) 90.01 (0.80) 88.97 (0.99)	89.98 (0.79) 88.35 (1.56) 91.05 (0.77) 91.36 (0.76) 91.26 (0.75) 91.75 (0.81)	1.75 (− 0.071; 3.56)^b^
QALY		2.68 (0.026)	2.70 (0.026)	0.024 (− 0.050; 0.097)
QALY discounted		2.64 (0.025)	2.66 (0.026)	0.023 (− 0.049; 0.095)
Healthcare costs				
Primary care Hospital care Emergency care Admission Day care Medication Home care Total healthcare costs Total healthcare costs discounted		978 (99) 2479 (311) 226 (40) 2 (2) 262 (125) 474 (32) 734 (209) 5209 (530) 5074 (515)	839 (76) 2472 (421) 201 (51) 163 (163) 4 (3) 492 (35) 533 (211) 4531 (594) 4414 (579)	− 139 (− 370; 83) − 8 (− 866; 938) − 26 (− 138; 100) 161 (− 6; 514) − 258 (− 574; − 68) 18 (− 67; 110) − 205 (− 787; 368) − 678 (− 2129; 803) − 660 (− 2078; 781)
Lost productivity costs		4313 (952)	1008 (350)	− 3305 (− 5028; − 1739)
Total societal costs		9521 (1114)	5539 (707)	− 3982 (− 6238; − 1731)
Total societal costs discounted		9295 (1088)	5400 (690)	− 3895 (− 6113; − 1712)

*SE* standard error, *95*% *CI* 95% confidence interval, *QALY* quality-adjusted life year, *EORTC QLQ*-*C30* European organization for research and treatment for cancer quality of life questionnaire

^a^Uncertainty around cost differences estimated using the non-parametric bootstrap with 5000 replications (bias-corrected intervals). The presented effect and cost differences are unadjusted

^b^Overall effect over time (36 months)

Multiple imputation model consisted of variables that differed at baseline, were related to missing data or were associated with the outcome: patient number, hospital, gender, diagnosis, age, stadium of tumour, location of tumour, differentiation grade of tumour, body mass index, costs of home care at baseline, costs of absenteeism at baseline, hospital costs at baseline, medication costs at baseline and primary care costs at baseline. The imputation procedure was stratified for treatment arm

**Table 3:** Cost-effectiveness results

Outcome	ΔC (95% CI)^a^	ΔE (95% CI)	ICER	CE plane			
Main analysis				NE	SE	SW	NW
EORTC QLQ-C30 summary score	− 2759 (− 4855; − 557)^c^	1.33 (− 0.049; 3.15)^b^	− 2073	1%	92%	7%	0%
QALYs	− 2759 (− 4855; − 557)^c^	− 0.021 (− 0.083; 0.040)^c^	129,164	0%	24%	75%	1%
Complete case analysis (SA1)							
EORTC QLQ-C30 summary score	− 4046 (− 8893; − 52)^c^	2.21 (0.71; 3.62)^b^	− 1832	3%	97%	0%	0%
QALYs	− 4046 (− 8893; − 52)^c^	− 0.023 (− 0.099; 0.056)^c^	178,551	2%	26%	71%	1%
Healthcare perspective (SA2)							
EORTC QLQ-C30 summary score	199 (− 1115; 1643)^c^	1.75 (− 0.044; 3.54)^b^	114	60%	38%	0%	2%
QALYs	199 (− 1115; 1643)^c^	− 0.021 (− 0.083; 0.040)^c^	− 9296	10%	14%	24%	52%
Undiscounted analysis (SA3)							
EORTC QLQ-C30 summary score	− 2816 (− 4996; − 564)^c^	1.75 (− 0.044; 3.54)^b^	− 1612	1%	96%	3%	0%
QALYs	− 2816 (− 4996; − 564)^c^	− 0.021 (− 0.084; 0.041)^c^	131,134	0%	24%	75%	1%
Unadjusted analysis (SA4)							
EORTC QLQ-C30 summary score	− 3895 (− 6060; − 1693)^c^	1.75 (− 0.044; 3.54)^b^	− 2230	0%	97%	3%	0%
QALYs	− 3895 (− 6060; − 1693)^c^	0.023 (− 0.048; 0.094)	− 167,574	0%	74%	26%	0%
Per protocol analysis (SA5)							
EORTC QLQ-C30 summary score	− 2279 (− 4786; 313)^c^	2.33 (0.35; 4.30)^b^	− 979	5%	94%	1%	0%
QALYs	− 2279 (− 4786; 313)^c^	− 0.013 (− 0.079; 0.052)^c^	169,659	1%	33%	62%	4%
Subgroup tumour stage 1 (SA6)							
EORTC QLQ-C30 summary score	− 3296 (− 7037; 243)^c^	0.85 (− 2.23; 3.93)^b^	− 3892	3%	68%	28%	1%
QALYs	− 3296 (− 7037; 243)^c^	0.051 (− 0.052; 0.15)^c^	− 65,267	3%	81%	15%	1%
Subgroup tumour stages 2 and 3 (SA7)							
EORTC QLQ-C30 summary score	− 2275 (− 5221; 362)	2.24 (0.028; 4.45)^b^	− 1016	6%	92%	2%	0%
QALYs	− 2275 (− 5221; 362)	− 0.064 (− 0.14; 0.0095)^c^	35,652	0%	4%	90%	6%

*95*% *CI* 95% confidence interval, *CE plane* cost-effectiveness plane, *ICER* incremental cost-effectiveness ratio, *NE* north-east quadrant, *NW* north-west quadrant, *SE* south-east quadrant, *SW* south-west quadrant, *QALY* quality-adjusted life year, *EORTC QLQ*-*C30* European organization for research and treatment for cancer quality of life questionnaire

^a^Uncertainty around cost differences estimated using the non-parametric bootstrap with 5000 replications (bias-corrected intervals). The presented cost differences are unadjusted

^b^The difference in EORTC QLQ-C30 summary score represent an overall effect time (36 months), which means that a correction for repeated observations took place

^c^The regression model for costs was adjusted for utility and EORTC score at baseline. The regression model for QALYs was adjusted for baseline utility

### Cost-effectiveness analyses

**Fig. 1:**
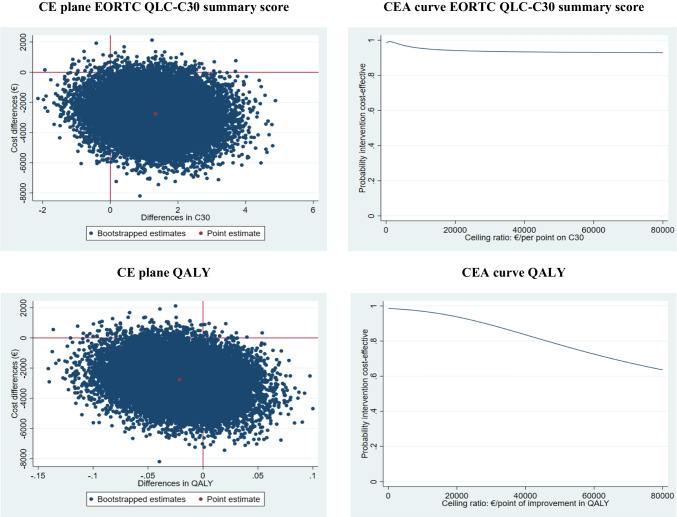
Cost-effectiveness figures. *CE plane* = cost-effectiveness plane, *CEA curve* cost-effectiveness acceptability curve, *QALY* quality-adjusted life year, *EORTC QLQ*-*C30* European organization for research and treatment for cancer quality of life questionnaire

The difference in QLQ-C30 summary score between the GP- and surgeon-led group was 1.33 points, indicating that over time GP-led care resulted in 1.33 points more improvement on the QLQ-C30 summary score more than surgeon-led care (see Table 3). This difference was not statistically significant (95% CI − 0.049; 3.15). The ICER for QLQ-C30 summary score was − 2073, indicating that GP-led care is dominant over surgeon-led care. The CE-plane (Fig. 1) shows that the majority of the bootstrapped cost-effect pairs is situated in the south-east quadrant of the plane, confirming a larger effect on the QLQ-C30 summary score and lower costs in the GP-led group as compared to the surgeon-led group. The CEA curve (Fig. 1) shows that the probability that GP-led care is cost-effective in comparison to surgeon-led care is 0.99, 0.99 and 0.95 at willingness-to-pay values of 0, 1000 and 10,000 € per point improvement in the QLQ-C30 summary score, respectively.

### Cost-utility analyses

The difference in QALYs between the GP- and surgeon-led group was − 0.021, which was not statistically significant (95% CI − 0.083; 0.040) (see Table 3). The ICER for QALYs was 129,164, indicating that €129,164 is saved in the GP-led group in comparison with the surgeon-led group while 1 QALY is lost. This large ICER is caused by the relatively small difference in QALYs between the groups. The CE-plane (Fig. 1) shows that the bootstrapped cost-effect pairs are mainly located in the south-west quadrant confirming the smaller effects and lower costs in the GP-led group as compared to the surgeon-led group. The CEA curve (Fig. 1) shows that the probability that GP-led care is cost-effective in comparison with surgeon-led care is 0.99, 0.94 and 0.78 at willingness-to-pay values of 0, 20,000 and 50,000 € per QALY gained, respectively.

### Sensitivity analyses

Restricting the cost-effectiveness analysis to complete cases only (SA1) and performing the analysis without discounting (SA3) gives relatively similar results compared to the main analysis (Table 3). However, performing the analysis from a healthcare perspective instead of societal perspective (i.e. excluding lost productivity costs) (SA2) shows that GP-led care was more costly than surgeon-led care (€199) instead of being cost-saving. However, this cost difference was not significant (95% CI − €1115; €1643). Consequently, it notably changed the probability that GP-led care is cost-effective in comparison with surgeon-led group. At willingness-to-pay values of 0, 20,000 and 50,000 € per QALY gained the probabilities were 0.38, 0.28 and 0.26, respectively. The unadjusted analysis (SA4) showed larger cost savings and differences in QLQ-C30 summary score. Also, the difference in QALYs (non-significant) became a positive difference compared to a negative difference in the main analysis. This means that in term of QALYs, GP-led care is cost-effective compared to surgeon-led care as it is less costly and more effective. The per protocol analysis, which only included the patients who had received care as intended by randomisation (SA5), resulted in a significant increase in QLQ-C30 summary score in the GP-led versus surgeon-led group compared to the main analysis (i.e. 2.33 points; 95% CI 0.35; 4.30). Performing the analysis on patients with stage 1 cancer resulted in larger cost savings, a non-significant increase in QALYs and a smaller non-significant increase in QLQ-C30 summary score compared to the main analysis. In the analysis on patients with stages II/III cancer, the effects were amplified, but the cost savings were smaller.

## Discussion

We performed an economic evaluation from a societal perspective alongside the randomised I CARE study. Total societal costs were significantly lower in the GP-led versus surgeon-led group (mean difference of − €3895; 95% CI − €6113; − €1712). The largest difference between the two groups was found for lost productivity costs (− €3305; 95% CI − €5028; − €1739). For the QLQ-C30 summary score, the main outcome of the study, GP-led care was dominant over surgeon-led care (i.e. less expensive, but more effective). However, for QALYs, GP-led care was not cost-effective.

### Comparison to existing literature

Costs of a consultation with a GP are substantially lower than of an outpatient consultation at the hospital (approximately €37 versus €89 euros in the year 2022 in the Netherlands) [5]. It is therefore not unexpected that survivorship care by a GP is cost-saving in comparison to care by a specialist. However, cost savings were mostly due to lost productivity costs, which was somewhat unexpected. Shortly after randomisation, 50 patients dropped-out, of which most patients were randomised to their GP [16]. Potential selective dropout may explain why lost productivity was the main contributor to the difference in total societal costs. Patients who experience ongoing problems or symptoms after surgery may prefer to remain in specialist care. Previous studies also concluded that survivorship care by a GP is less costly, though the level of evidence was low [11, 12]. To our knowledge, only one previous randomised trial has investigated cost-effectiveness of transferring follow-up after colon cancer from the specialist to the GP [39]. In this Norwegian trial, there were no significant differences in QoL between the trial arms, and therefore a cost minimization analysis was performed. The authors showed that GP organised follow-up was associated with societal cost savings over a 24-month period (converted into euros there was a mean difference of €2.073). Similarly, a randomised trial among breast cancer patients showed cost savings when follow-up was provided in primary care instead of in secondary care (mean difference of €1.985) [40]. Both trials also demonstrated cost savings relating to lost productivity, which is in line with our results. Here, we provide additional evidence that GP-led survivorship care is cost-effective compared to surgeon-led care. These results are highly needed to help control the increasing healthcare costs for cancer survivors [15].

### Implications for practice and research

First, it is important to note that the results for QLQ-C30 summary score and QALYs were contradictory. Even though QLQ-C30 summary score improved over time (1.33), QALYs decreased (− 0.021). In our study, the difference in QALYs was very small, not statistically significant and below the minimally important QALY difference often used in cancer research (difference of 0.06 to 0.07) [41]. In this study, we used the three-level version of the EQ-5D, but the recently developed five-level version of the EQ-5D could have been more sensitive than the three-level version to pick up changes in general QoL [42]. Because there were no important differences in general QoL outcomes, it might be more informative to look at disease-specific QoL outcomes. The QLQ-C30 summary score is a composite score of 13 scales, and is likely to be more sensitive to pick up changes in QoL of colon cancer survivors [22].

From an economic point of view, survivorship care by a GP seems preferable to care by a surgeon and is likely to help improve sustainability and affordability of care. However, when it comes to evaluating and implementing new models of care, it is also important to consider other factors, including patient and physician preferences [15]. I CARE participants also mentioned barriers to engaging GPs in cancer care [43, 44]. Barriers included, among others, a lack of knowledge and experience of GPs and the amount of extra work for GPs. Another concern was the timeliness and appropriateness of follow-up testing by GPs, resulting in potential problems with delayed diagnosis of recurrences [45]. GPs will therefore require additional time, additional personnel and financial compensation to provide this type of care for all cancer patients.

### Strengths and limitations

This economic evaluation was performed alongside a large randomised-controlled trial comparing colon cancer survivorship care by a GP to care by a surgeon. This allowed for the prospective collection of cost and effect data. Compared to two previous trials, this study has the longest follow-up duration (3 years versus 24 and 18 months) [39, 46]. The study had a pragmatic design, in which patients and physicians were free to organise care as they thought appropriate, thereby mimicking actual clinical practice as closely as possible. We used a societal perspective, meaning that many relevant costs were included in the analyses and potential cost shifts between sectors or budgets can be identified. Multiple imputation was used to impute missing observations, decreasing the magnitude of potential bias due to selective dropout.

There are also limitations that need to be addressed. First, the study was performed within the Dutch healthcare system. All Dutch citizens are required to register with a GP, who acts as a gatekeeper to hospital-based care. Differences in healthcare systems around the world limit the transferability of the applicability and costs of the new model of care. Even though the baseline characteristics were evenly distributed among the trial arms, there were slightly more patients with stage I tumours in the GP- versus surgeon-led group (33 versus 42%). Because these patients typically require less follow-up consultations than patients with stages II/III tumours, it may also explain some of the differences in costs. Subgroup analyses did show larger cost savings of GP-led care in stage I tumours, even though it was not significant. Finally, to assess healthcare utilisation and productivity losses, we used retrospective self-reported questionnaires which may have caused recall bias. However, we assume that this type of bias is equally distributed over both trial arms and therefore does not affect the differences between groups.

## Conclusions

From a societal perspective, GP- versus- surgeon-led colon cancer survivorship care results in significantly lower costs. For disease-specific QoL, GP-led care is likely to be cost-effective (i.e., less expensive, but more effective). For general QoL, it is not. Besides cost-effectiveness, it is important to think about the extra time and workload for GPs. These factors should be taken into consideration when discussing a possible transfer of survivorship care from the surgeon to the GP.

## Data Availability

At the end of study, data can be made available, after anonymization, on request to the corresponding author, taking into account possible national and international legal restrictions.

## References

[1] Arnold M, Sierra MS, Laversanne M, Soerjomataram I, Jemal A, Bray F. Global patterns and trends in colorectal cancer incidence and mortality. Gut. 2017;66(4):683–91. 26818619 10.1136/gutjnl-2015-310912

[2] Hewitt M, Greenfield S, Stovall E. From cancer patient to cancer survivor: lost in transition. Washington, DC: National Academies Press; 2006.

[3] Qaderi SM, Dickman PW, de Wilt JHW, Verhoeven RHA. Conditional survival and cure of patients with colon or rectal cancer: a population-based study. J Natl Compr Canc Netw. 2020;18(9):1230–7. 32886900 10.6004/jnccn.2020.7568

[4] The Dutch Ministry of Health, Welfare and Sports (VWS) [Internet]. Accessed 11th of May 2022. [Available from: https://www.vzinfo.nl/dikkedarmkanker/zorguitgaven].

[5] Hakkaart-van Roijen L, Van der Linden N, Bouwmans CAM, et al. Costing manual: methodology of costing research and reference prices for economic evaluations in healthcare. Diemen: Dutch Healthcare Institute; 2016.

[6] Brandenbarg D, Roorda C, Groenhof F, Havenga K, Berger MY, de Bock GH, et al. Increased primary health care use in the first year after colorectal cancer diagnosis. Scand J Prim Health Care. 2014;32(2):55–61. 24931639 10.3109/02813432.2014.929811 PMC4075017

[7] Heins M, Schellevis F, Rijken M, van der Hoek L, Korevaar J. Determinants of increased primary health care use in cancer survivors. J Clin Oncol. 2012;30(33):4155–60. 23071230 10.1200/JCO.2012.41.9101

[8] Emery JD, Shaw K, Williams B, Mazza D, Fallon-Ferguson J, Varlow M, et al. The role of primary care in early detection and follow-up of cancer. Nat Rev Clin Oncol. 2014;11(1):38–48. 24247164 10.1038/nrclinonc.2013.212

[9] Health Council of the Netherlands. Follow-up in oncology—identify objectives, substantiate actions. 2007; publication no. 2007/10.

[10] Noteboom EA, Perfors IA, May AM, Stegmann ME, Duijts SF, Visserman EA, et al. GP involvement after a cancer diagnosis; patients’ call to improve decision support. BJGP Open. 2021;5(1):bjgpopen20X101124. 33172850 10.3399/bjgpopen20X101124 PMC7960515

[11] Vos JAM, Wieldraaijer T, van Weert H, van Asselt KM. Survivorship care for cancer patients in primary versus secondary care: a systematic review. J Cancer Surviv. 2020;15(1):66–76. 32815087 10.1007/s11764-020-00911-w PMC7822798

[12] Hoeg BL, Bidstrup PE, Karlsen RV, Friberg AS, Albieri V, Dalton SO, et al. Follow-up strategies following completion of primary cancer treatment in adult cancer survivors. Cochrane Database Syst Rev. 2019;2019(11):425. 10.1002/14651858.CD012425.pub2 PMC6870787 31750936

[13] Duineveld LA, Wieldraaijer T, van Asselt KM, Nugteren IC, Donkervoort SC, van de Ven AW, et al. Improving care after colon cancer treatment in The Netherlands, personalised care to enhance quality of life (I CARE study): study protocol for a randomised controlled trial. Trials. 2015;16:284. 26112050 10.1186/s13063-015-0798-7 PMC4499213

[14] Vos JAM, Duineveld LAM, Wieldraaijer T, Wind J, Busschers WB, Sert E, et al. Effect of general practitioner-led versus surgeon-led colon cancer survivorship care, with or without eHealth support, on quality of life (I CARE): an interim analysis of 1-year results of a randomised, controlled trial. Lancet Oncol. 2021;22(8):1175–87. 34224671 10.1016/S1470-2045(21)00273-4

[15] Jefford M, Howell D, Li Q, Lisy K, Maher J, Alfano CM, et al. Improved models of care for cancer survivors. The Lancet. 2022;399(10334):1551–60. 10.1016/S0140-6736(22)00306-3 PMC9009839 35430022

[16] Duineveld LAM, Vos JAM, Wieldraaijer T, Donkervoort SC, Wind J, van Weert H, et al. Recruitment challenges to the I CARE study: a randomised trial on general practitioner-led colon cancer survivorship care. BMJ Open. 2021;11(8): e048985. 34429313 10.1136/bmjopen-2021-048985 PMC8386209

[17] National Guideline Colorectal Carcinoma (CRC) [Internet]. Available from: https://www.oncoline.nl/colorectaalcarcinoom [Accessed 10th of June 2020].

[18] Castor EDC. Castor Electronic Data Capture 2019 [Internet]. Available from: https://www.castoredc.com [Accessed 27th of August 2019].

[19] Qaderi SM, Galjart B, Verhoef C, Slooter GD, Koopman M, Verhoeven RHA, et al. Disease recurrence after colorectal cancer surgery in the modern era: a population-based study. Int J Colorectal Dis. 2021;36(11):2399–410. 33813606 10.1007/s00384-021-03914-w PMC8505312

[20] Aaronson NK, Ahmedzai S, Bergman B, Bullinger M, Cull A, Duez NJ, et al. The European Organization for Research and Treatment of Cancer QLQ-C30: a quality-of-life instrument for use in international clinical trials in oncology. J Natl Cancer Inst. 1993;85(5):365–76. 8433390 10.1093/jnci/85.5.365

[21] Brooks R. EuroQol: the current state of play. Health Policy. 1996;37(1):53–72. 10158943 10.1016/0168-8510(96)00822-6

[22] Giesinger JM, Kieffer JM, Fayers PM, Groenvold M, Petersen MA, Scott NW, et al. Replication and validation of higher order models demonstrated that a summary score for the EORTC QLQ-C30 is robust. J Clin Epidemiol. 2016;69:79–88. 26327487 10.1016/j.jclinepi.2015.08.007

[23] Lamers LM, Stalmeier PF, McDonnell J, Krabbe PF, van Busschbach JJ. Measuring the quality of life in economic evaluations: the Dutch EQ-5D tariff. Ned Tijdschr Geneeskd. 2005;149(28):1574–8. 16038162

[24] iMTA Productivity and Health Research Group. Manual iMTA Medical Cost Questionnaire (iMCQ) [Internet]. Available from: https://www.imta.nl/questionnaires/imcq/publications/ [Accessed 15th of May 2022].

[25] The Dutch Ministry of Health, Welfare and Sports (VWS) - Dutch Healthcare Institute [Internet]. Available from: https://www.medicijnkosten.nl/zoeken [Accessed 18th of May 2022].

[26] van Roijen L, Essink-Bot ML, Koopmanschap MA, Bonsel G, Rutten FF. Labor and health status in economic evaluation of health care. The Health and Labor Questionnaire. Int J Technol Assess Health Care. 1996;12(3):405–15. 8840661 10.1017/S0266462300009764

[27] van Buuren S, Groothuis-Oudshoorn K. Mice: multivariate imputation by chained equations in R. 2011. J Stat Softw. 2011;45(3):67. 10.18637/jss.v045.i03

[28] Faria R, Gomes M, Epstein D, White IR. A guide to handling missing data in cost-effectiveness analysis conducted within randomised controlled trials. Pharmacoeconomics. 2014;32(12):1157–70. 25069632 10.1007/s40273-014-0193-3 PMC4244574

[29] White IR, Royston P, Wood AM. Multiple imputation using chained equations: issues and guidance for practice. Stat Med. 2011;30(4):377–99. 21225900 10.1002/sim.4067

[30] Rubin DB. Multiple imputation for nonresponse in surveys. Wiley, New York; 1987. p. xxix, 258.

[31] El Alili M, van Dongen JM, Goldfeld KS, Heymans MW, van Tulder MW, Bosmans JE. Taking the analysis of trial-based economic evaluations to the next level: the importance of accounting for clustering. Pharmacoeconomics. 2020;38(11):1247–61. 32729091 10.1007/s40273-020-00946-y PMC7546992

[32] Twisk JW. Applied multilevel analysis: a practical guide for medical researchers. Cambridge University Press; 2006.

[33] Twisk JW. Applied longitudinal data analysis for epidemiology: a practical guide. Cambridge University Press; 2013.

[34] Twisk JW. Analysis of data from randomized controlled trials. A practical guide Cham. Springer; 2021.

[35] Manca A, Hawkins N, Sculpher MJ. Estimating mean QALYs in trial-based cost-effectiveness analysis: the importance of controlling for baseline utility. Health Econ. 2005;14(5):487–96. 15497198 10.1002/hec.944

[36] Fenwick E, O’Brien BJ, Briggs A. Cost-effectiveness acceptability curves—facts, fallacies and frequently asked questions. Health Econ. 2004;13(5):405–15. 15127421 10.1002/hec.903

[37] Hoch JS, Dewa CS. Advantages of the net benefit regression framework for economic evaluations of interventions in the workplace: a case study of the cost-effectiveness of a collaborative mental health care program for people receiving short-term disability benefits for psychiatric disorders. J Occup Environ Med. 2014;56(4):441–5. 24662952 10.1097/JOM.0000000000000130

[38] Karpenko AW, Geenen JW, Vreman RA, Hovels A. The introduction of a threshold for the icer and the implications for reimbursement of drugs in the Dutch healthcare system. Value in Health. 2017;20(9):A671. 10.1016/j.jval.2017.08.1645

[39] Augestad KM, Norum J, Dehof S, Aspevik R, Ringberg U, Nestvold T, et al. Cost-effectiveness and quality of life in surgeon versus general practitioner-organised colon cancer surveillance: a randomised controlled trial. BMJ Open. 2013;3(4):e002391. 23564936 10.1136/bmjopen-2012-002391 PMC3641467

[40] Grunfeld E, Gray A, Mant D, Yudkin P, Adewuyi-Dalton R, Coyle D, et al. Follow-up of breast cancer in primary care vs specialist care: results of an economic evaluation. Br J Cancer. 1999;79(7–8):1227–33. 10098764 10.1038/sj.bjc.6690197 PMC2362235

[41] Pickard AS, Neary MP, Cella D. Estimation of minimally important differences in EQ-5D utility and VAS scores in cancer. Health Qual Life Outcomes. 2007;5:70. 18154669 10.1186/1477-7525-5-70 PMC2248572

[42] Hernandez Alava M, Wailoo A, Grimm S, Pudney S, Gomes M, Sadique Z, et al. EQ-5D-5L versus EQ-5D-3L: the impact on cost effectiveness in the United Kingdom. Value Health. 2018;21(1):49–56. 29304940 10.1016/j.jval.2017.09.004

[43] Vos JAM, de Best R, Duineveld LAM, van Weert HCPM, van Asselt KM. Delivering colon cancer survivorship care in primary care; a qualitative study on the experiences of general practitioners. BMC Primary Care. 2022;23(1):13. 35172743 10.1186/s12875-021-01610-w PMC8761520

[44] Vos JA, van Miltenburg VE, Beverdam FH, van Weert HC, van Asselt KM. Patient experiences of GP-led colon cancer survivorship care: a Dutch mixed-methods evaluation. Br J Gen Pract. 2023;73(727):e115–23. 36316164 10.3399/BJGP.2022.0104 PMC9639600

[45] Vos JAM, Sert E, Busschers WB, Duineveld LAM, Wieldraaijer T, Wind J, et al. Detection of colon cancer recurrences during follow-up care by general practitioners versus surgeons. J Natl Cancer Inst. 2023. 10.1093/jnci/djad019 10.1093/jnci/djad019 PMC10165489 36715623

[46] Wattchow DA, Weller DP, Esterman A, Pilotto LS, McGorm K, Hammett Z, et al. General practice vs surgical-based follow-up for patients with colon cancer: randomised controlled trial. Br J Cancer. 2006;94(8):1116–21. 16622437 10.1038/sj.bjc.6603052 PMC2361245

